# COVID-19, Grippewellen und ökonomische Aktivität — die Perspektive der Wirkungsanalyse

**DOI:** 10.1007/s10273-020-2655-x

**Published:** 2020-05-20

**Authors:** Adalbert Winkler

**Affiliations:** International and Development Finance, Frankfurt School of Finance and Management, Sonnemannstrasse 9-11, 60314 Frankfurt a. M., Deutschland

**Keywords:** C31, I15, I18

## Abstract

Die öffentliche Diskussion zur Corona-Krise nimmt an Schärfe zu. Dabei stehen die Letalitätsrate sowie die Wirkungen staatlicher Maßnahmen in Hinblick auf ihre epidemiologischen und ökonomischen Wirkungen im Mittelpunkt. Der Streit ließe sich klären, wenn man an randomisierte, kontrollierte Studien angelehnte Wirkungsanalysen durchführen könnte. Dies ist mangels Kontrollgruppe aber nicht möglich. So ist eine weiter zunehmende Polarisierung zu erwarten, gerade wenn die Todeszahlen niedrig bleiben.

## Abstract (en)

The German debate about the policy implications of the coronavirus becomes increasingly heated given different views on the mortality rate from SARS CoV-2, as well as the epidemiological and economic impact of lockdown measures imposed by the government. Impact analysis applying features of randomised control trials would provide clear evidence. However, this is currently unavailable due to a lack of an appropriate control group of countries without measures. Thus, polarisation is likely to rise if observed mortality rates remain low.

Die Corona-Krise ist zum zentralen Gegenstand gesellschaftspolitischer Diskussionen geworden. Drei Fragen stehen dabei im Mittelpunkt:

1. Wie ist die Wirkung von SARS-CoV-2, also wie gefährlich ist das Coronavirus?

2. Wie wirken die Maßnahmen, das Virus einzudämmen?

3. Sind diese Maßnahmen verhältnismäßig, also wie groß sind ihre negativen Wirkungen auf die ökonomische Aktivität[1] im Vergleich zu einem Szenario, in dem sich die Krankheit COVID-19 ungebremst ausbreitet?

Die herrschende Sichtweise (Ferguson et. al., 2020) ist, dass das Virus 1) bei ungebremstem Verlauf sehr gefährlich ist, also zu Hunderttausenden Toten führen würde, und 2) soziale Distanzierung die Ausbreitung wirkungsvoll eindämmt, gleichzeitig aber 3) mit starken Einbrüchen der ökonomischen Aktivität einhergeht. Dennoch ist es selbst ökonomisch sinnvoll, solche Maßnahmen staatlich zu verordnen, wenn freiwillige Maßnahmen das Coronavirus nicht (mehr) entscheidend bremsen und die Menschen aus Angst ökonomische Aktivitäten noch deutlicher und länger herunterfahren als ohne Lockdown.

Diese Sichtweise hat in den ersten Wochen der Pandemie in Deutschland viel öffentliche Zustimmung erfahren. Die Ereignisse in Italien, Spanien und Frankreich bestätigten die Gefährlichkeit des Virus, jene in Großbritannien und den USA signalisierten, dass bei zu spätem staatlichem Handeln die ökonomische Tätigkeit ohnehin drastisch einbricht. Entsprechend gering erschienen die volkswirtschaftlichen Kosten der staatlich angeordneten Maßnahmen, während hohe „Erträge“ in Form von vermiedenen Infektionen und Toten winkten. So kam es auch: Die Zahl der Toten blieb in Deutschland begrenzt, ohne dass dafür im Vergleich zu anderen Staaten mit einem stärkeren Einbruch der ökonomischen Aktivität „bezahlt“ werden musste.

Es gab und gibt aber auch eine andere Sicht, nämlich dass a) COVID-19 eine geringe Sterblichkeit aufweist, die einer mittelschweren Influenza ähnelt, b) die staatlich verordneten Maßnahmen das Virus nicht wirksam eindämmen, und c) diese Maßnahmen aber erhebliche negative ökonomische Wirkungen aufweisen und daher sofort zu beenden sind (Homburg, 2020a, 2020b). Diese Sichtweise hat in Deutschland in den letzten Wochen an Aufmerksamkeit gewonnen. Denn bei insgesamt moderaten Infektions- und Todeszahlen, die nicht mehr durch katastrophale Bilder aus anderen Ländern ergänzt werden, wirkt die wirtschaftliche Entwicklung für viele immer bedrohlicher. Zusammen

* Dieser Beitrag wurde durch die Diskussion zu COVID-19 in der von Carl Christian von Weizsäcker ins Leben gerufenen Makrogruppe motiviert. Zudem danke ich Christian Breuer, Benjamin Born, Gerhard Illing, Holger Sandte, Oliver Stein und Eva Terberger für wertvolle Anregungen und Kommentare.

1 Es gibt natürlich auch negative Konsequenzen in anderen gesellschaftlichen Bereichen, die hier aber nicht thematisiert werden sollen.

**Prof. Dr. Adalbert Winkler** ist Professor für International and Development Finance an der Frankfurt School of Finance & Management in Frankfurt am Main.

senkt dies die Bereitschaft, staatliche Anti-Corona-Maßnahmen als das kleinere Übel zu akzeptieren, weil die Menschen nicht mehr erfahren, dass COVID-19 gefährlich ist, dass die Maßnahmen wirken und es sich lohnt, den ökonomischen Preis dafür zu zahlen.

Die Methodik der Wirkungsanalyse erklärt dieses Verhalten. Weil die ökonomischen Konsequenzen der Bekämpfung von COVID-19 über staatliche Maßnahmen so drastisch erscheinen, verlangen die Menschen – wie vor der Einführung eines lebensrettenden Medikaments mit starken Nebenwirkungen – nach dem methodischen „Goldstandard" der Wirkungsanalyse, um sich der Gefährlichkeit des Virus, der Wirksamkeit der Maßnahmen und ihrer ökonomischen Verhältnismäßigkeit zu vergewissern: Sie verlangen nach Evidenz, wie ihn randomisierte, kontrollierte Studien (RCT) liefern, also nach einem Vergleich zwischen der Situation jetzt und jener, die in mit Deutschland ähnlichen Kontrollländern zu beobachten wäre, wenn keine Maßnahmen gegen COVID-19 ergriffen worden wären und daher die ungebremste Wirkung von COVID-19 auf Sterblichkeit und die ökonomische Entwicklung erfahrbar wäre. Denn so ließen sich die Fragen des gesundheitspolitischen Erfolgs und der ökonomischen Verhältnismäßigkeit staatlich angeordneter Maßnahmen in gleicher Weise auf der Basis von Fakten klären, wie die Fragen nach den Erfolgsaussichten und den Nebenwirkungen einer Chemotherapie bei einer Krebsbehandlung.

Das Problem ist: Es gibt solche Kontrollländer nicht (mehr). COVID-19-Todesfälle unter den Bedingungen, wie sie bei einer Grippewelle herrschen, lassen sich im Mai 2020 nicht (mehr) beobachten. Die herrschende Sichtweise muss daher für ihre Politikempfehlungen allein auf der Basis kontrafaktischer, nicht-beobachtbarer Zahlen werben, wie sie sich aus wissenschaftlichen Studien ergeben, die die Gefährlichkeit von COVID-19 und die Wirksamkeit der Maßnahmen nachweisen (Flaxman et al., 2020) sowie über die ökonomischen Konsequenzen ungebremster Pandemien informieren (Jordá et al., 2020). Diese sind aber für viele Menschen kaum verständlich. Zudem gibt es auch im Corona-Streit Wissenschaftler, die der herrschenden Sichtweise widersprechen. Der Verweis auf das Wissen der „Experten" ist daher nicht eindeutig. Gerade weil viele um die nackte Existenz bangen, steigt der Wunsch, konkret und eindeutig zu sehen, dass sich die Opfer lohnen und verhältnismäßig sind, und nicht nur mit kontrafaktischen, also nicht beobachtbaren Daten – so viele Tote würde es gegeben, so stark würde die Wirtschaft einbrechen, wenn die Regierungen nicht handeln würden – abgespeist zu werden.

In dieses Vakuum stößt die alternative Sichtweise, indem sie die beobachtbaren, niedrigen COVID-19-Sterbefälle und die Talfahrt der Wirtschaft als Beleg für die Richtigkeit ihrer Position interpretiert: Staatliche Maßnahmen haben nach dieser Sicht keine Wirkung auf die Entwicklung der COVID-19-Sterbefälle, dafür aber eine desaströse Wirkung auf die ökonomische Aktivität. Das Fehlen einer nach RCT-Methoden durchführbaren Wirkungsanalyse führt also dazu, dass wir einer immer stärkeren Polarisierung der öffentlichen Diskussion entgegengehen, und zwar je geringer die Infektions- und Todeszahlen und je stärker die ökonomischen Verwerfungen bei weiterhin restriktiven staatlichen Maßnahmen ausfallen und je stärker die ökonomischen Verwerfungen allein den Maßnahmen von Bund und Ländern zugeschrieben werden.

## Wirkungsanalysen und COVID-19-Streit

Der COVID-19-Streit wäre schnell entschieden, wenn für Deutschland COVID-19-Daten sowie Informationen zur ökonomischen Entwicklung vorliegen würden, ohne dass Bund und Länder Maßnahmen getroffen hätten (counterfactual). Es ist unmittelbar einsichtig, dass dies grundsätzlich nicht möglich ist. Die Methodik der Wirkungsanalyse zeigt dennoch eine Möglichkeit, die Frage „was wäre gewesen, wenn ..." zu beobachten. Dazu sind zu Deutschland passende Kontrollländer notwendig, die auf staatlich verordnete Maßnahmen gegen COVID-19 verzichtet haben. Dann lässt sich – wie in der Medizin bei der Wirkungsanalyse von Medikamenten – die RCT-Methodik anwenden, um die Wirkung von COVID-19 auf Sterblichkeit und ökonomische Entwicklung ohne staatliche Maßnahmen festzustellen. In den Wirtschaftswissenschaften ist diese Methode mit der Vergabe des Nobelpreises 2019 an die Entwicklungsökonomen Banerjee, Duflo und Kremer einem größeren Publikum bekannt geworden.

Das Problem ist, dass diese als „Goldstandard" der Wirkungsanalyse (Glennerster und Takavarasha, 2013, vgl. auch Kasten 1) bekannte Methode zur Beantwortung der kontrovers diskutierten Fragen um COVID-19 aus zwei Gründen nicht zur Verfügung steht:

- Kein Land weist in Bezug auf andere Störgrößen, die die Ausbreitung und Mortalität von COVID-19 unabhängig von staatlichen Maßnahmen bestimmen, eine identische Verteilung zu Deutschland auf. Ein ernst zu nehmender Ländervergleich muss also für solche Störgrößen kontrollieren, z. B. für unterschiedliche Bevölkerungsdichten, Einkommensungleichheiten und Sensitivitäten der Bevölkerung hinsichtlich der Gefahr von Viren. Erste Ergebnisse zeigen, dass dann staatlich verordneten Maßnahmen eine positive Wirkung zur Eindämmung des Virus zugeschrieben werden kann (Hatzius et al., 2020). Allerdings stehen solche Untersuchungen grundsätzlich unter dem Vorbehalt, dass sie andere Variablen, die die Verbreitung des Virus wo-

Kasten1

**Die RCT-Wirkungsanalyse – dargestellt am Beispiel COVID-19**

Für Länder, die Maßnahmen gegen COVID-19 ergreifen (T), und für Länder, die keine Maßnahmen gegen COVID-19 ergreifen (C), lassen sich vier Zustände definieren (Ausführungen lehnen sich eng an Armendáriz und Morduch (2010)):

1. $(Y_1|T)$ = Infektions- bzw. Todeszahlen, die in Ländern eintreten, die Maßnahmen treffen. Deutschland gehört zu diesen Ländern. Die Zahlen, die das Robert Koch-Institut (RKI) oder die Johns-Hopkins-Universität (JHU) für Deutschland veröffentlichen, entsprechen $(Y_1|T)$.

2. $(Y_0|T)$ = Infektions- bzw. Todeszahlen, die in den Ländern, die Maßnahmen ergriffen haben, eingetreten wären, hätten sie diese nicht ergriffen. Dies sind die Zahlen, die RKI und JHU für Deutschland veröffentlichen würden, wenn es keine Maßnahmen gegen COVID-19 gegeben hätte. $(Y_0|T)$ steht also für jene Zahlen, aus denen man ablesen könnte, ob die Krankheit COVID-19 wirklich so gefährlich ist wie behauptet. Auf der Basis solcher Zahlen wäre dann auch leichter zu entscheiden, ob die getroffenen Maßnahmen verhältnismäßig sind. Allerdings gibt es diese Zahlen nicht, weil Deutschland Maßnahmen ergriffen hat. $(Y_0|T)$ steht also für das „counterfactual“, die kontrafaktischen Infektions- und Todeszahlen.

3. $(Y_0|C)$ = Infektions- bzw. Todeszahlen, die in Ländern eintreten, die keine Maßnahmen treffen. Solche Länder gibt es (mittlerweile) nicht (mehr). Bestenfalls für den Beginn der Krise, also solange COVID-19 unbekannt war bzw. solange Bevölkerungen und Regierungen auf die Nachrichten aus China nicht mit Verhaltensänderungen und Politikmaßnahmen reagierten, können die JHU-Zahlen für diese Länder näherungsweise als Beobachtungen von $(Y_0|C)$ interpretiert werden.

4. $(Y_1|C)$ = Infektions- bzw. Todeszahlen, die in den Ländern, die keine Maßnahmen ergriffen haben, eingetreten wären, wenn sie Maßnahmen ergriffen hätten. Wegen (3) kann es solche Länder nicht geben. Allerdings: In den Ländern, die spät reagiert und eine hohe Zahl von Toten zu beklagen haben, z. B. in Großbritannien, wird über $(Y_1|C)$ diskutiert, weil sie die Infektions- und Todeszahlen darstellen, die eingetreten wären, wenn früher reagiert worden wäre, also das Kontrafaktische darstellen.

In einer RCT-Studie ließen sich die kontrafaktischen Zahlen für Deutschland beobachten, weil unter der Bedingung, dass C- und T-Länder statistisch vergleichbar sind, Deutschland und die Kontrollgruppe die gleichen Erwartungswerte (E) in beiden Zuständen aufweisen. Es gilt also:

$E[Y_0|T] = E[Y_0|C]$, und $E[Y_1|T] = E[Y_1|C]$

Sofern es also statistisch vergleichbare Länder gäbe, die Maßnahmen gegen COVID-19 ergriffen und die solche Maßnahmen nicht ergriffen haben, ließen sich $E[Y_1|T]$ und $E[Y_0|C]$ beobachten und damit die Wirksamkeit der Maßnahmen feststellen. Denn es gilt

(1) $$E[Y_1|T] - E[Y_0|C] = E[Y_1|T] - E[Y_0|T] + \{ E[Y_0|T] - E[Y_0|C]\}$$

Da der Erwartungswert linear ist, lässt sich Gleichung (1) schreiben als:

(2) $$E[Y_1|T] - E[Y_0|C] = E[Y_1 - Y_0|T] + \{ E[Y_0|T] - E[Y_0|C]\}$$

Der erste Term auf der rechten Seite von Gleichung (2) stellt dar, worüber die Menschen in Deutschland gerne Gewissheit haben möchten: Die Differenz zwischen den Infektions- und Todeszahlen, wenn Bevölkerung und Regierung in Deutschland nicht handeln würden (bzw. nicht gehandelt hätten) – also das Kontrafaktische zu den beobachtbaren Infektions- und Todeszahlen. Der zweite Term auf der rechten Seite ist gleich null, weil T- und C-Länder statistisch identisch sind. Entsprechend wird Gleichung (2) zu:

(3) $$E[Y_1|T] - E[Y_0|C] = E[Y_1 - Y_0|T]$$

Gleichung (3) besagt, dass die (erwartete) Differenz der Infektions- und Todeszahlen zwischen Ländern, die handeln, und Ländern, die nicht handeln, gleich der Differenz zwischen den tatsächlichen und kontrafaktischen Infektions- und Todeszahlen in Ländern sind, die – wie Deutschland – Maßnahmen ergriffen haben. Die gleiche Logik gilt, um die ökonomischen Wirkungen der staatlich angeordneten Maßnahmen zweifelsfrei bestimmen zu können.

möglich beeinflussen, nicht berücksichtigen (omitted variable bias). Zu nennen wären z. B. unterschiedliche Kapazitäten der Gesundheitssysteme, die Zahl von Massenveranstaltungen, und kulturell bedingte Unterschiede in der sozialen Distanzierung vor COVID-19 (Umarmungen, Händeschütteln etc.). Es kann also nicht ausgeschlossen werden, dass die Wirkung staatlich verordneter Maßnahmen in einem Modell, das alle Einflussfaktoren berücksichtigt, schwächer ausfallen oder gar nicht zu beobachten sind.

- Es bedarf Länder, die keine Maßnahmen gegen COVID-19 ergriffen haben, die sich also wie in Grippezeiten verhalten haben. Solche Länder gibt es aber nicht mehr. Es gab sie bestenfalls zu Beginn der Krise, also als COVID-19 noch gar nicht bekannt war bzw. Regierungen die Nachrichten aus China als weitgehend irrelevant für das eigene Verhalten zur Kenntnis nahmen (vgl. Kasten 1).

## Wenn die Kontrollgruppe fehlt …

Die Tatsache, dass mittlerweile alle Länder staatliche Maßnahmen gegen die Ausbreitung von COVID-19 ergriffen haben, hat zur Folge, dass die von den Virologen für den Fall einer ungebremsten Entwicklung vorhergesagte Sterblichkeit X nicht beobachtet werden kann. $Y_0|C$ bleibt unbekannt. Empirisch lässt sich nur die Sterblichkeit von COVID-19 in Ländern mit staatlichen Maßnahmen beobachten, also $Y_1|T$. Unbestritten ist, dass diese Zahl in allen Ländern derzeit weit unter X liegt.

Aus der herrschenden Sichtweise zeigt dies den Erfolg der Maßnahmen: Wie vorhergesagt haben sie die Sterblichkeit von COVID-19 erheblich reduziert (Ferguson et al., 2020; Flaxman et al., 2020). Mit einem kontrafaktischen Experiment belegen lässt sich dies aber wegen der fehlenden Beobachtbarkeit von X nicht. Zweifelsfrei ist nur, dass es mittlerweile Länder gibt, in denen die Zahl der mit COVID-19 in Verbindung gebrachten Todesfälle, $Y_1|T$, (erheblich) über denen einer mittelschweren Grippe G liegt[2] und Übersterblichkeit herrscht (Euromomo, 2020; Wu et al., 2020). Selbst wenn also von der Annahme ausgegangen wird, dass die staatlichen Maßnahmen unwirksam sind, d. h. $Y_1|T = Y_0|C$, kann die These, dass COVID-19 nicht schlimmer ist als eine mittelschwere Influenza, für diese Länder nicht mehr aufrechterhalten werden.

In Deutschland sind die Todeszahlen aber weiterhin moderat; auch Übersterblichkeit lässt sich bisher nicht feststellen (Statistisches Bundesamt, 2020), was die herrschende Meinung auf den Erfolg der Maßnahmen zurückführt. Die zweite Sichtweise interpretiert aber die Zahlen anderer Länder als Ergebnis des Einflusses von Störgrößen, die die Sterblichkeit von COVID-19 dort erhöhen würden, in Deutschland aber nicht gegeben seien. Folglich seien sie für die Diskussion über die Gefährlichkeit von COVID-19 in Deutschland wenig oder gar nicht relevant. Zudem belege $Y_1|T > G$ nicht, dass die staatlichen Maßnahmen richtig und wirkungsvoll seien. Denn um dies eindeutig (und) politisch vermittelbar zu belegen, müsste man $Y_0|C$ kennen. Auch aus den exponentiellen Wachstumsraten zu Beginn der Krise lässt sich nicht zweifelsfrei ableiten, dass $Y_0|C = X$ ist, und dies nur dank der staatlichen Maßnahmen verhindert wurde. Denn möglich wäre es, dass die Zahlen auch ohne die Maßnahmen zurückgegangen wären, und zwar gerade dann, wenn man gleichzeitig die These vertritt, dass die mit COVID-19 verbundene Sterblichkeit vergleichsweise gering sei. Und da eine passende Kontrollgruppe fehlt, lässt sich diese These nicht mit Verweis auf beobachtbare Zahlen, die die tatsächliche Höhe von X ausweisen, eindeutig widerlegen.[3]

2 Zwischen 2010 und 2019 lag in den USA die Zahl der für das Gesamtjahr geschätzten Grippetoten nicht einmal über der schon jetzt erreichten Zahl von COVID-19-Toten, also 79.000 (Stand: 10. Mai 2020). In Schweden waren in den letzten drei Jahren (2016 bis 2019) laut den jeweiligen Berichten der Gesundheitsbehörde weniger Grippetote (713/1.021/505) zu beklagen als COVID-19-Tote (3.225, Stand: 10. Mai 2020).

3 Allerdings können Schätzungen zum Verlauf der Infektions- und Todeszahlen auf der Basis logistischer Funktionen, die in einem frühen Stadium der Epidemie vorgenommen wurden, als alternative Prognosen ($X_{Alt}$) von X gelten. So prognostiziert Homburg (2020a) auf dieser Basis Obergrenzen für die Infektions- bzw. Todeszahlen, die in Deutschland in Zusammenhang mit COVID-19 eintreten werden, in Höhe von 123.000 bzw. ca. 6.150. Die aktuellen Werte der Johns-Hopkins-Universität liegen aber bei 171.704 bzw. 7.549 (Stand: 10. Mai 2020). Selbst wenn die staatlichen Maßnahmen unwirksam gewesen sein sollten, weist COVID-19 schon jetzt ca. 40 % bzw. 22 % höhere Infektions- bzw. Todesraten auf, als es die Daten Anfang April für den gesamten Pandemieverlauf in Deutschland vermuten ließen. Auch für die USA weist dieser Ansatz erhebliche Prognosefehler auf, da die für die Gesamtheit (!) der Epidemie vorhergesagten Zahlen von der Realität bereits nach wenigen Tagen (!) übertroffen wurden (Piper, 2020). Solche starken Abweichungen sind darin begründet, dass gerade zu Beginn des Prozesses die beobachtbaren Wachstumsraten mit stark unterschiedlichen Verläufen der Funktion im weiteren Verlauf vereinbar sind (Avery et al., 2020; Cochrane, 2020). Luo (2020) sieht Prognosefehler auch in einer schwankenden Intensität und Wirkung von freiwilligen und staatlichen Maßnahmen über die Zeit hinweg begründet, also abhängig von einem Faktor, über den heftig gestritten wird. Schließlich lässt sich mit dieser Methode nichts darüber sagen, ob eine zweite Infektionswelle droht oder nicht. Dies hängt davon ab, ob und wie stark die Reproduktionszahl wieder über 1 steigt, mit oder ohne Maßnahmen. Dennoch lassen sich solche Prognosen von der zweiten Sichtweise in der öffentlichen Diskussion argumentativ gut verwenden, weil der Prognosefehler im Vergleich zu den Zahlen, die von den Virologen für den ungebremsten Verlauf von COVID-19 genannt wurden, klein ist, also $X_{Alt}$ deutlich näher an $Y|T$ liegt als X. Dass $X_{Alt}$ und X unter völlig anderen Bedingungen prognostiziert wurden, spielt kaum eine Rolle, solange darüber nicht gesprochen wird bzw. diese anderen Bedingungen, konkret: die mittlerweile ergriffenen Maßnahmen, erfolgreich als irrelevant für die Einschätzung der Prognosegüte dargestellt werden.

Tabelle 1

**Deutschland und Schweden: Kennzahlen zur Corona-Pandemie**

| | Intensität staatlicher Maßnahmen gegen COVID-19[1] | COVID-19-Sterblichkeit pro 100.000 Einwohner[2] | Bevölkerungsdichte (pro $km^2$ Landfläche), (2018) | Gini-Koeffizient | Prognostizierte Wachstumsrate des BIP[3] in % | Offenheitsgrad (Summe aus Exporten und Importen in % des BIP, 2018) | Anteil des internationalen Tourismus an den Gesamtexporten (in %, 2018) |
|---|---|---|---|---|---|---|---|
| Deutschland | 32 | 7,1 | 237,7 | 31,9 (2016) | -7,0 | 88,67 | 3,22 |
| Schweden | 21 | 22,5 | 25,0 | 28,8 (2017) | -6,8 | 89,07 | 5,91 |

[1] Mittelwert der von der Universität Oxford angegebenen täglichen Indexwerte zwischen dem 1.1.2020 und 28.4.2020. [2] Grunddaten von Oxford Martin School, Stand: 28.4.2020 [3] Internationaler Währungsfonds (IWF): Prognose für 2020 des World Economic Outlook vom April 2020.

Quelle: eigene Zusammenstellung und Berechnungen, Weltbank (sofern nicht anders vermerkt).

Gleichzeitig wird immer deutlicher, dass COVID-19 die bei weitem schwerste Rezession der Nachkriegszeit auslöst. Damit verschiebt sich die Diskussion immer mehr auf die Frage der Verhältnismäßigkeit staatlich angeordneter Maßnahmen. In dieser Diskussion haben die Vertreter der zweiten Sichtweise einen strukturellen Vorteil, weil sie mit beobachtbaren Zahlen argumentieren können. Jeder Tag, an dem die Infektions- und Todeszahlen sinken bzw. auf niedrigem, Grippe-ähnlichem Niveau verharren, kann als Bestätigung der Grippethese, jeder Tag, an dem die ökonomischen Verwerfungen zunehmen, als Beleg für die Unverhältnismäßigkeit der Maßnahmen gewertet werden.

## Ökonomische Wirkungen der Maßnahmen zur Eindämmung von COVID-19

Die Diskussion über die Verhältnismäßigkeit staatlich angeordneter Maßnahmen wird suggerierend oft so geführt, als seien allein diese Maßnahmen für den Einbruch der Wirtschaft verantwortlich. Dies ist aber nicht der Fall, denn zum einen wirken sich auch freiwillige Maßnahmen der sozialen Distanzierung sowie freiwillige und staatliche Maßnahmen in anderen Ländern negativ auf die hiesige wirtschaftliche Entwicklung aus. Die Methodik der Wirkungsanalyse zeigt aber auch hier, dass sich mangels geeigneter Kontrollgruppe und aufgrund von Antizipations- und Spillover-Effekten an RCT angelehnte Wirkungsanalysen nicht durchführen lassen (Glennerster und Takavashara, 2013). Erneut lässt sich also die Wirkung staatlich angeordneter Maßnahmen nicht eindeutig ermitteln.

Dies lässt sich illustrieren, wenn man Deutschland mit Schweden vergleicht, das in der öffentlichen Diskussion oft als Kontrollland verwendet wird, weil es in geringerem Maße auf staatlich angeordnete soziale Distanzierung gesetzt hat als Deutschland. So ist der Mittelwert eines Indexwertes, der die Intensität staatlicher Maßnahmen gegen COVID-19 für viele Länder erfasst, für den Zeitraum vom 1.1.2020 bis zum 28.4.2020 in Deutschland (32) höher als in Schweden (21, vgl. Tabelle 1). Gleichzeitig weist Schweden aber trotz deutlich niedrigerer Bevölkerungsdichte und etwas gleicherer Einkommensverteilung ungefähr dreimal so viele COVID-19-Todesfälle pro 100.000 Einwohner auf wie Deutschland. Sie sind aber nach wie vor deutlich unter X.[4] Daher könnte man aus ökonomischer Sicht den Verzicht auf stärkere staatlich angeordnete Maßnahmen als sinnvoll werten, sofern Schweden dadurch die volkswirtschaftlichen Kosten im Vergleich zu Deutschland senken könnte.

Die beobachtbaren Daten sprechen jedoch zunächst dagegen. Die ökonomischen Kosten sind in beiden Ländern praktisch gleich. So prognostiziert der Internationale Währungsfonds (IWF) für 2020 ein Wirtschaftswachstum von -6,8 % für Schweden und von -7,0 % für Deutschland.[5] Sofern die IWF-Prognose einigermaßen zutrifft, und die restriktiveren Maßnahmen in Deutschland zusätzliche Leben retten, wären die ökonomischen Kosten dieser Rettung also verhältnismäßig gering bis gar nicht vorhanden.

Allerdings sind Schweden und Deutschland hinsichtlich der ökonomischen Wirkungen von Anti-Corona-Maßnahmen ebenfalls nicht strukturgleich. So kann Schweden von den Maßnahmen anderer Länder trotz annähernd gleichem Offenheitsgrad stärker betroffen sein als Deutschland (spillovers), weil beispielsweise der Tourismus dort einen deutlich höheren Stellenwert aufweist als in Deutschland (vgl. Tabelle 1). Der Tourismus ist wegen der Reisebeschränkungen praktisch vollkommen zum

4 Möglich ist auch, dass in Schweden wegen der liberaleren Politik eine Herdenimmunität schon weiter vorangeschritten ist, sodass die derzeit niedrigeren Todeszahlen in Deutschland (relativ zur Gesamtbevölkerung) lediglich aussagen, dass eine größere Zahl von Toten bisher nur aufgeschoben wurde (Stern, 2020).

5 Dies wurde auch in den Medien aufgegriffen, vgl. Focus (2020). In der Frühjahrsprognose der EU-Kommission beträgt der Unterschied zwischen den für 2020 prognostizierten Wachstumsraten 0,4 Prozentpunkte.

Erliegen gekommen, während der Welthandel als Folge der Pandemie „nur" stark rückläufig ist. Trotz nahezu gleicher Effekte aller Maßnahmen insgesamt, der in Form der IWF-Prognosen „beobachtbar" ist, könnte die liberalere schwedische Anti-Corona-Politik also einen positiven Effekt auf die ökonomische Entwicklung in Schweden haben, den auch Deutschland durch entsprechende Lockerungen erzielen könnte. Umgekehrt wäre aber auch möglich, dass die Menschen in Schweden aus Angst vor dem Virus stärkere freiwillige Maßnahmen der sozialen Distanzierung ergreifen als in Deutschland („Antizipationseffekte"). Folglich könnte in Schweden ein Teil der negativen volkswirtschaftlichen Effekte des staatlich angeordneten Lockdowns, die wir in Deutschland vielleicht haben, durch größere negative Effekte freiwilliger Maßnahmen ersetzt worden sein. Dies würde erklären, warum für Schweden trotz liberalerer Politik ein ähnlich starker Rückgang des Bruttoinlandsprodukts zu erwarten ist.[6] Erneut zeigt sich: Da die Bedingungen für eine RCT-Studie nicht gegeben sind, lassen sich Wirkungen staatlicher Anti-Corona-Maßnahmen auf die volkswirtschaftliche Entwicklung in jedem Land nicht eindeutig ermitteln.

## Schlussfolgerungen aus der Wirkungsanalyse für die Corona-Krise

Die zur Bekämpfung von COVID-19 getroffenen Maßnahmen gehen mit stark negativen Wachstumsraten der Realeinkommen einher. Viele Menschen, die von der ökonomischen Krise hart getroffen sind, fragen daher, ob die staatlichen Maßnahmen verhältnismäßig sind. Aber auch jene, denen es noch gut geht, treibt die Sorge um, dass sie bald belastet werden, um die Kosten der Krise gerecht zu verteilen. Beide Gruppen möchten daher eindeutig bestätigt bekommen, dass sich die ökonomischen Opfer lohnen. Dies gilt vor allem dann, wenn die Corona-Todeszahlen moderat sind.

Die Methodik der Wirkungsanalyse zeigt, dass diese Eindeutigkeit nicht erreicht werden kann, weil mangels geeigneter Kontrollgruppe nicht beobachtet werden kann, wie sich COVID-19 und die ökonomische Aktivität ohne staatliche Maßnahmen entwickelt hätten. Damit lässt sich eine RCT-ähnliche Analyse nicht anwenden. Wir sehen nur die Entwicklungen in Ländern, die sich in Bezug auf die Ausbreitungsbedingungen von COVID-19 sowie in Dringlichkeit und Intensität von freiwillig oder staatlich angeordneten Maßnahmen zur Eindämmung des Virus unterscheiden. So lässt sich weder der Effekt der Maßnahmen auf die Entwicklung von COVID-19 noch der Effekt der Maßnahmen auf die ökonomische Entwicklung eindeutig bestimmen. Klar ist daher bisher nur, dass in vielen Ländern COVID-19 schlimmer als eine mittelschwere Grippe ist.

Aus diesen Überlegungen lässt sich schließen, dass in der öffentlichen Diskussion in Deutschland eine vergleichsweise restriktive Anti-Corona-Politik immer schwieriger durchzusetzen sein wird, weil die Todeszahlen so moderat sind und die in der Wissenschaft herrschende Sichtweise daher den bisherigen Forschungsstand zu COVID-19 und die sich daraus ableitenden Politikimplikationen nicht auf der Basis beobachtbarer Zahlen überzeugend kommunizieren kann.[7] Denn angesichts des fehlenden Kontrafaktischen wird es für immer mehr Menschen zu einer Glaubensfrage, wie tödlich das Coronavirus wirklich ist, wie sehr die restriktiven Maßnahmen wirken und ob der zu zahlende ökonomische Preis verhältnismäßig ist.[8] Zur Beantwortung dieser Frage suchen die Menschen nach Rat, nicht nur, aber auch aus der Wissenschaft. Und hier haben die Vertreter der zweiten Sichtweise – das Virus ist nicht so gefährlich, die Maßnahmen wirken nicht, die Maßnahmen haben hohe ökonomische Kosten – den Vorteil mit dem scheinbar Offensichtlichen, nämlich Beobachtbaren, argumentieren zu können, während die Vertreter der herrschenden Sichtweise – COVID-19 ist gefährlich und die Maßnahmen wirken – verstärkt auf Studien und das durch die Maßnahmen verhinderte Kontrafaktische verweisen müssen. In der öffentlichen Debatte haben sie daher einen schweren Stand, solange die Gegenseite fast unwidersprochen den Eindruck erwecken kann, die mit COVID-19 verbundenen volkswirtschaftlichen Kosten würden vor allem auf die staatlichen Maßnahmen zurückgehen und nicht vermittelt wird, dass sich vergleichbare Kosten auch als Folge freiwilliger Maßnahmen und starker

6 In seiner Einschätzung der volkswirtschaftlichen Kosten staatlicher Maßnahmen von Bund und Ländern weist das Ifo-Institut explizit auf die Möglichkeit hin, dass die staatlichen Maßnahmen zumindest teilweise freiwillige Maßnahmen ersetzen (Dorn et al., 2020). Die Wirkungen der Maßnahmen anderer Länder werden dagegen nicht thematisiert, gleichzeitig aber als kontrafaktisches Szenario die Ifo-Prognose vom Dezember 2019, also eine florierende Weltwirtschaft, gewählt. Angesichts der Exportorientierung der deutschen Wirtschaft legt dies nahe, dass die Studie eher eine Wirkungsanalyse des Auftretens von Corona als des Shutdowns darstellt. Dagegen bemüht sich die Konjunkturforschungsstelle Zürich (KOF-Institut) für die Schweiz, die Auslandseinflüsse explizit zu berücksichtigen (Rathke et al., 2020). Je nach Szenario wird diesen vom Staat gar nicht zu beeinflussenden Einflüssen ein Anteil zwischen 38 % und 71 % der mit Corona einhergehenden volkswirtschaftlichen Kosten in der Schweiz zugeschrieben.

7 Christian Drosten spricht in diesem Zusammenhang vom „Präventions-Paradox", weil die Kommunikationsprobleme der herrschenden Sichtweise eine direkte Folge ihrer erfolgreichen Umsetzung sind, vgl. z. B. Schneider (2020).

8 Ähnlich wird es in Ländern sein, die erhebliche negative wirtschaftliche Folgen der Pandemie und aller getroffenen Maßnahmen zu beklagen haben, und nicht über die notwendigen Strukturen verfügen, diese Folgen sozial verträglich absichern zu können.

Spillovers, ausgelöst von noch strikteren Maßnahmen bei Handelspartnern, ergeben können.

Damit legt die Methodik der Wirkungsanalyse nahe, dass es selbst aus der Perspektive der herrschenden Sichtweise sinnvoll sein kann, bei den administrativen Maßnahmen nun im Zweifelsfall – also nicht bei der Frage von Großveranstaltungen etc. – etwas zu sehr zu lockern, und dafür stärker auf Mahnung und Warnung, also auf Freiwilligkeit bei klaren Rahmenbedingungen zu setzen, sowie Kapazitäten aufzubauen, um einer möglichen zweiten Infektionswelle besser begegnen zu können. Denn ein zu starkes Beharren auf restriktiven Maßnahmen zur Vermeidung einer zweiten Infektionswelle ist gerade dann nicht durchzuhalten, wenn a) dieses Beharren Erfolg zeigt, also mit weiterhin niedrigen Infektions- und Todeszahlen einhergeht, b) kein anderes Land, das von den Menschen als zu Deutschland passendes Kontrollland empfunden wird, eine zweite Welle erlebt und c) gleichzeitig die ökonomischen Hiobsbotschaften zunehmen, die vor allem der restriktiven Anti-Corona-Politik zugeschrieben werden. Denn dann steigt die Wahrscheinlichkeit, dass es zu einer überstürzten Öffnung kommt. Wenn ich die herrschende Sichtweise zu COVID-19 richtig verstehe, ist es aber das Szenario einer überstürzten Öffnung, das es auf jeden Fall zu vermeiden gilt.
